# In Silico Insights into the Mechanism of Action of Epoxy-α-Lapachone and Epoxymethyl-Lawsone in *Leishmania* spp.

**DOI:** 10.3390/molecules26123537

**Published:** 2021-06-10

**Authors:** Juliana Figueiredo Peixoto, Adriane da Silva Oliveira, Patrícia Queiroz Monteiro, Luiz Filipe Gonçalves-Oliveira, Valter Viana Andrade-Neto, Vitor Francisco Ferreira, Franklin Souza-Silva, Carlos Roberto Alves

**Affiliations:** 1 Laboratório de Biologia Molecular e Doenças Endêmicas, Instituto Oswaldo Cruz, Fundação Oswaldo Cruz, Rio de Janeiro 21040-900, Brazil; jufpeixoto@gmail.com (J.F.P.); adrianecirrus@gmail.com (A.d.S.O.); or patricia.monteiro@ioc.fiocruz.br (P.Q.M.); luizfilipeol07@gmail.com (L.F.G.-O.); 2 Laboratório de Bioquímica de Tripanossomatídeos, Instituto Oswaldo Cruz, Fundação Oswaldo Cruz, Rio de Janeiro 21040-900, Brazil; valter@ioc.fiocruz.br; 3 Departamento de Tecnologia Farmacêutica, Faculdade de Farmácia, Universidade Federal Fluminense, Niterói 24241-002, Brazil; vitorferreira@id.uff.br; 4 Centro de Desenvolvimento Tecnológico em Saúde, Fundação Oswaldo Cruz, Rio de Janeiro 21040-900, Brazil; 5 Faculdade de Ciências Biológicas e da Saúde, Universidade Iguaçu, Avenida Abílio Augusto Távora, 2134, Dom Rodrigo, Nova Iguaçu CEP 26260-045, Brazil

**Keywords:** *Leishmania* spp., leishmaniases, epoxy-α-lapachone, epoxymethyl-lawsone, molecular docking

## Abstract

Epoxy-α-lapachone (Lap) and Epoxymethyl-lawsone (Law) are oxiranes derived from Lapachol and have been shown to be promising drugs for Leishmaniases treatment. Although, it is known the action spectrum of both compounds affect the *Leishmania* spp. multiplication, there are gaps in the molecular binding details of target enzymes related to the parasite’s physiology. Molecular docking assays simulations were performed using DockThor server to predict the preferred orientation of both compounds to form stable complexes with key enzymes of metabolic pathway, electron transport chain, and lipids metabolism of *Leishmania* spp. This study showed the hit rates of both compounds interacting with lanosterol C-14 demethylase (−8.4 kcal/mol to −7.4 kcal/mol), cytochrome c (−10.2 kcal/mol to −8.8 kcal/mol), and glyceraldehyde-3-phosphate dehydrogenase (−8.5 kcal/mol to −7.5 kcal/mol) according to *Leishmania* spp. and assessed compounds. The set of molecular evidence reinforces the potential of both compounds as multi-target drugs for interrupt the network interactions between parasite enzymes, which can lead to a better efficacy of drugs for the treatment of leishmaniases.

## 1. Introduction

Leishmaniases are a group of diseases caused by more than 20 species of the *Leishmania* genus and are classified as neglected tropical diseases. The infection is endemic in approximately 98 countries and 350 million people are at risk of getting infected [1]. The disease occurs after promastigote forms inoculation in humans through the bite of sandflies of the genera *Lutzomyia* (New World) and *Phlebotomus* (Old World). In the mammalian host, these parasites differentiate in amastigote forms within cells, mainly macrophages, and affect the skin (cutaneous Leishmaniasis-CL) and/or oropharyngeal mucosa and cartilage (mucocutaneous Leishmaniasis-ML). However, some species can infect cells of tissues and internal organs, such as liver, spleen, and bone marrow, causing visceral Leishmaniasis (VL), a form of unfavorable prognosis and difficult parasitological cure [2,3].

Since the 1940s, pentavalent antimonials, such as meglumine antimoniate and sodium stibogluconate, are considered the main drugs used to treat all clinical forms of leishmaniases, especially in the New World. In contraindication cases, intolerance or low therapeutic response to pentavalent antimonials, second-line drugs are used, such as Amphotericin B, pentamidine, miltefosine, and paromomycin [1]. Currently, two pentavalent antimonials varieties are being used sodium stibogluconate (Pentostam) and meglumine antimoniate (Glucantime). Their effectiveness and toxicity effects are related to Sb^5+^ content. Despite of their structural differences, they are considered therapeutically similar, even though the Pentostam formulation contains almost 20% more Sb^5+^ than Glucantime [1]. Despite the longevity of this therapeutic regimen, pentavalent antimonials are often associated with a high incidence of mild to moderate adverse effects, such as musculoskeletal pain, gastrointestinal disorders, headache, and anorexia. In addition, several patients have presented severe clinical conditions due the treatment, such as cardiac, hepatic, and pancreatic toxicity [4]. On one hand, antimonials seem to inhibit glycolytic enzymes and components of the fatty acid oxidation pathway. On the other hand, they had not shown potential to inhibit the hexose monophosphate pathway and the citric acid cycle. This data is based on in vitro experiment exposure of *Leishmania* (*Leishmania*) *mexicana* to Pentostam, resulting in a decreasing dose-dependent cell viability and CO_2_ production from glucose and palmitate from parasite cultures, leading to a depletion of intracellular ATP levels [5]. There is evidence that Sb^3+^ inhibits trypanothione reductase (TR) activity, an enzyme essential for the survival of the parasite within macrophages, in *L.* (*L.*) *donovani* [6]. Furthermore, it was found that Sb^3+^ binds, with high affinity, to the active site of the TR of *L.* (*L.*) *infantum*, inhibiting its enzymatic activity [7].

Amphotericin B deoxycholate is a well-known polyene antibiotic and antifungal obtained from *Streptomyces nodosus*, and it has been reported in in vitro and in vivo experiments to be effective against both promastigote and amastigote forms of *Leishmania* [8]. Attempting to reduce the side effects of amphotericin B have led to the development of lipid formulations of this drug. Currently, three lipid formulations based on amphotericin B are available on the market: liposomal amphotericin B, amphotericin B lipid complex, and amphotericin B colloidal dispersion [9,10,11]. These formulations present similar effectiveness of the deoxycolate salt, being significantly less toxic, but because of their high cost, their use in developing countries is still restricted [12]. Miltefosine, a hexadecylphosphocholine, was originally developed as an oral antineoplastic agent (for skin cancer treatment). It was approved after several clinical studies, becoming the first oral leishmaniasis treatment [13]. The most common adverse effects with the use of miltefosine are diarrhea and vomiting, occurring in more than 30% of the cases. Due to its teratogenic effects, it is contraindicated during pregnancy [14]. Paromomycin (aminosidine) is the only aminoglycoside with leishmanicidal activity and can be used for visceral and cutaneous forms. As the drug is poorly absorbed by the oral route, a parenteral formulation for the treatment of VL and a topical formulation for the treatment of LC has been developed [15,16,17].

**Figure 1:**
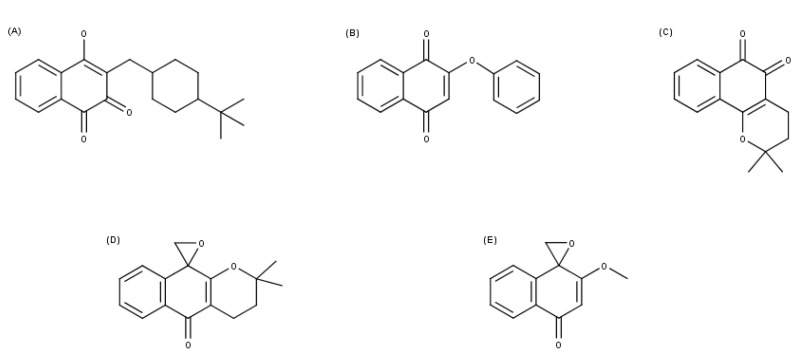
Structural formulas of naphthoquinone and its derivatives. (**A**) Buparvaquone (3-[(4-tert-butylcyclohexyl)methyl]-4-hydroxynaphthalene-1,2-dione); (**B**) B6 (2-phenoxy-1,4-naphthoquinone); (**C**) β-lapachone (2H-Naphtho[1,2-b]pyran-5,6-dione,3,4-dihydro-2,2-dimethyl); (**D**) Epoxy-α-lapachone (2,2-Dimethyl-3,4-dihydro-spiro[2H-naphtho[2,3-b]pyran-10,2′-oxirane]-5(10H)-one); and (**E**) Epoxymethyl-lawsone (2-Methyl-4H-spiro-[naphthalene-1,20-oxiran]-4-one).

Although, these compounds are still being used after decades and have been proven to be effective, several adverse effects and the high toxicity increase the need for the search of new therapies for these diseases. Naphthoquinones, a particular group of quinones, and their derivatives have a wide spectrum of biological activities and represent a group of interesting compounds for chemotherapy. Among these compounds, we highlighted two oxiranes, epoxy-α-lapachone (Lap: 2,2-dimethyl-3,4-dihydrospiro[benzo[g]chromene-10,20-oxiran]-5(2H)-one)) and epoxymethyl-lawsone (Law: 2-methyl-4H-spiro-[naphthalene-1,20-oxiran]-4-one), (Figure 1), which have been shown to have potential to be used in the treatment of leishmaniases [18,19,20,21,22].

These compounds are derivated from lapachol, a natural naphthoquinone that was originally isolated from heartwood of *Bignoniaceae* and *Verbanaceae* [23,24,25]. The high toxicity that Lapachol presents leads to the development of molecular derivatives. Lap and Law were generated through chemical modifications of the quinonoid center of α-lapachone and 2-hydroxy-1, 4-naphthoquinone (Lawsone), respectively, followed by formation of the epoxide ring [26,27].

It has already been reported that Lap can inhibit serine and cysteine proteinase activities in *Trypanosoma cruzi* and serino proteinase in *L.* (*L.*) *amazonensis* [19,28]. However, the mechanisms of action of Law remains unknown in the literature. It is only known that Lap can cross the plasma membrane of macrophages and acting directly killing amastigote forms of *L.* (*L.*) *amazonensis* [21]. Furthermore, there is a need for a better understanding of the mechanisms of action of both compounds. The characteristics of their mechanisms were not yet known until this present study.

In this work, molecular docking assays were conducted in comparison with other naphthoquinones derivatives previously assessed in vitro, such as 2H-Naphtho[1,2-b]pyran-5,6-dione,3,4-dihydro-2,2-dimethyl (β-lapachone), (3-[(4-tert-butylcyclohexyl)methyl]-4-hydroxynaphthalene-1,2-dione (Buparvaquone), and 2-phenoxy-1,4-naphthoquinone (B6), (Figure 1). A discussion of naphthoquinone derivatives molecular target-set *Leishmania* spp. related with cutaneous leishmaniases was initiated in the American continent. In silico prediction of the possible activities of Lap and Law against *L.* (*V.*) *braziliensis* and *L.* (*L.*) *amazonensis* was accessed targeting enzymes of metabolic pathways, electron transport chain, and lipid biosynthesis, reinforcing the mechanisms of action of both naphthoquinone derivatives in *Leishmania* spp.

## 2. Results and Discussion

The action of quinone derivatives over protein/enzyme targets from *Leishmania* spp. is related to their three-dimensional structure, which guides the interaction with the binding site in the target enzymes, leading to the beneficial or adverse effects of these compounds. Despite the knowledge accumulated in this broad spectrum of actions of naphthoquinone derivative compounds, there are still gaps concerning the molecular binding detail in the enzymes related to the *Leishmania* spp. physiology, mainly, enzymes from vital metabolic pathways of these parasites, e.g., energy metabolism, electron transport chain, and lipid biosynthesis. Thus, the motivation for this study was to add evidence in silico that cytochrome c, lanosterol C-14 demethylase, and glycosomal glyceraldehyde 3-phosphate dehydrogenase are targets for Lap and Law in *Leishmania* spp.

Quinones and derivative, with naphthoquinones among them, present in their structures two carbonyl groups in an unsaturated ring of six carbon atoms, located in ortho or para positions. The effects of these compounds are related to the ortho- and para-quinonoid center when they accept one and/or two electrons, constituting the redox cycle, forming ROS. This cycle causes cellular damage-accelerating intracellular hypoxia. Furthermore, among quinone action, the toxicity potential through DNA alkylation and the interaction with topoisomerases, bioactive enzymes involved in DNA duplication have been described in previous studies [29,30,31,32].

On the other hand, it is well known in the literature that α-lapachone, even though it is a naphthoquinone, would not be involved in the ROS production, and for this reason, it was believed that its derivatives would not have this biological effect either, especially on the parasite *T. cruzi* [33,34]. Indeed, the trypanocidal activity of these compounds might be related to another mechanism of action; and with the introduction of the oxirane ring in the quinonoid center, forming Lap, there was an increasing in the trypanocidal activity [34].

To elucidate the mechanism of action of Lap, as well as the other naphthoquinone derivatives, structural changes were proposed in the precursor molecule, α-lapachone and in the β-lapachone isomer, since it was known that the C ring of these molecules constitutes an important structural characteristic for biological activity, as well as the strong influence of the redox center, generating a series of compounds including Lap and Law [35].

Lap was tested against *T. cruzi* epimastigote forms and showed to be able to reduce its growth by inhibiting parasite serine proteinase, similarly to phenylmethylsulfonyl-fluoride (PMSF), a classic inhibitor of this type of enzyme [25]. Similar effects were observed in *L. (L.) amazonensis* promastigotes and amastigotes, since Lap inhibited the parasite serine proteinase activity, as the PMSF, aprotinin, and antipain inhibitors. Further studies conducted in silico indicated that Lap binds to a serine proteinase, Oligopeptidase B, and migth be a potential pharmacological target against *Leishmania* spp. [19]. Besides *Leishmania* spp. and *T. cruzi*, there is evidence that Lap acts as serine protease inhibitor in *Plasmodium falciparum* [36].

The potential of Lap and Law acting on proteins and/or key enzymes of the metabolic pathways of parasitic etiologic agents of human disease is still poorly understood. However, it is possible to draw a parallel of effects between Lap, Law, and other naphthoquinone derivatives, since these compounds share structural similarities in a common molecular skeleton, highlighting that the mechanism of action of these compounds may be based on the oxidation and reduction properties [37]. The study of different metabolic pathways, such as glycolysis pathway, fatty acid metabolism, and ergosterol biosynthesis, can be explored as chemotherapeutic targets in trypanosomatids [38,39,40]. The docking results presented here with Lap and Law showed favorable binding energy to the target enzymes, as well as Buparvaquone, compound B6 (2-phenoxy-1,4-naphthoquinone), and β-lapachone (Table 1).

**Figure 2:**
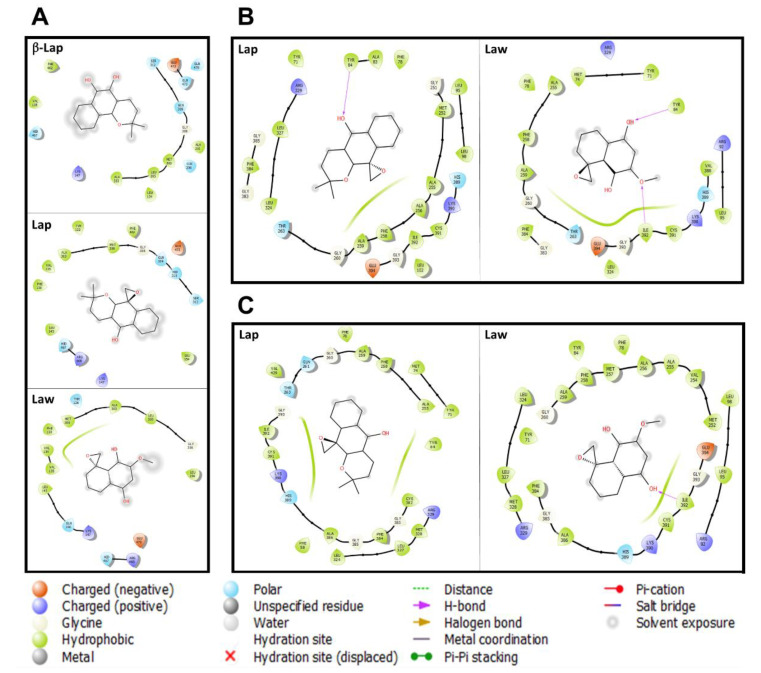
Molecular interaction of naphthoquinone derivatives with *Leishmania* spp. lanosterol C-14 demethylase. Docking assays were performed with (**A**) Lanosterol C-14 demethylase of *Coccidioides posadasii*; (**B**) *L. (L.) amazonensis*; (**C**) *L. (V.) braziliensis*. The 2D representation indicates the types of bonds that occur in Lanosterol C-14 demethylase with β-lapachone (2H-Naphtho[1,2-b]pyran-5,6-dione,3,4-dihydro-2,2-dimethyl-β-Lap), Epoxy-α-lapachone (2,2-dimethyl-3,4-dihydrospiro[benzo[g]chromene-10,20-oxiran]-5(2H)-one-Lap), and Epoxymethyl-lawsone (2-Methyl-4H-spiro-[naphthalene-1,20-oxiran]-4-one-Law).

The natural β-lapachone derivative has been shown to act on the fungal membrane of *Coccidioides posadasii* and *Histoplasma capsulatum*, affecting the permeability of the cytoplasmic membrane by reducing the ergosterol content [41]. In *Leishmania* spp., the lanosterol C-14 demethylase is the one of lipids metabolism enzyme, and numerous studies pointed to its biological role, showing that its inhibition alters membrane permeability, infectivity, and mitochondrial function [39,42,43,44]. In fact, the inhibition of different enzymes in the ergosterol biosynthesis pathway, such as HMG-CoA reductase, squalene epoxidase, lanosterol C-14 demethylase, and sterol C-24 methyltransferase, exhibit potent trypanocidal and leishmanicidal activities [45]. Therefore, docking molecular assays performed here with lanosterol C-14 demethylase, a cytochrome P450 monooxygenase (CYP51 gene family), were motivated for this central role in lipid biosynthesis, followed by previous description of β-lapachone inhibiting cytochrome P450 enzyme, CYP3A4, as well as the azoles (e.g., miconazole), lanosterol C-14 demethylase inhibitor group [46,47]. These findings indicate the potential action of naphthoquinones, as well as Lap and Law, on lipid biosynthesis that was reinforced in this study by molecular docking results (Figure 2). Possibly, both Lap and Law compounds assessed here may be inhibiting enzymes in the lipids metabolism because there are reports of inhibition of this pathway by β-lapachone, another naphthoquinone-derivate [41].

The molecular details of the *C. posadasii* Lanosterol C-14 demethylase binding site with β-lapachol did not show hydrogen bonds identified in the PDB structures (Table S1). However, there is a predominance of hydrophobic (*n* = 8), polar (*n* = 7), and charged (*n* = 1) amino acid residues in the binding site. Comparatively, Lap and Law occupy the same binding site that β-lapachol, maintaining their amino acid residues profiles of 69% and 50%, respectively. In this case, hydrophobic (*n* = 9), polar (n ≤ 5), and charged (*n* = 2) amino acid residues are involved in the binding site (Figure 2). Furthermore, data showed that both compounds have solvent exposure and the interaction with lanosterol C-14 demethylase occurs by hydrophobic interactions. The similarity in the way these compounds bind to the enzyme is represented by the score value (≃−8.0 kcal/mol; Table 1).

The docking between lanosterol C-14 demethylase of *L. (L.) amazonensis* and *L. (V.) braziliensis* with Lap and Law reflect different intraspecific and interspecific profiles. In *L. (L.) amazonensis*, the binding site from Lap and Law was composed by hydrophobic amino acid residues and both performed hydrogen bonds with TYR84, a bond presented in crystallized structures with TYR116 (Table S1). The difference between both compounds is the hydrogen bonding with ILE392 and a greater solvent exposure in lanosterol C-14 demethylase/Law complex. The contribution of these interactions can be confirmed by the bonding energy of Lap (−8.4 kcal/mol) and Law (−7.4 kcal/mol). The lanosterol C-14 demethylase binding site of *L. (V.) braziliensis* interacts predominantly with hydrophobic amino acid residues in both compounds. However, it was demonstrated that only Law has a hydrogen bond with ILE392. The presence of this bond has not contributed to Law better binding energy, compared to Lap. These differences in the energy values are related to the greater solvent exposure in lanosterol C-14 demethylase/Law complex (Figure 2).

Since the docking strategy performed here indicated the interaction of the enzyme lanosterol C-14 demethylase with Lap and Law; therefore, it was opportune to investigate the effect of the compounds on the inhibition of lipid biosynthesis. In fact, the inhibition of lipid biosynthesis in *L. (V.) braziliensis* promastigotes was confirmed here. The incubation of these compounds with promastigotes indicated that both of them can interfere in lipid biosynthesis profile and acquired by the parasite (Figure S1 and Appendix A).

The action of naphthoquinone derivatives on mitochondrial activity, and possibly against energy metabolism, seems to be a common and well-known mechanism on tumor cells, in *T. cruzi* and *L. (L.) amazonensis* [48,49,50]. Even the Lap derivative has been shown to be able to promote mitochondrial damage altering ion pumping flux of *L. (L.) amazonensis*. Lap can quickly cross the plasma membrane of the parasite, and can collapse mitochondrial membrane, release of cytochrome c into the cytosol [19].

In this context, naphthoquinones derivatives such as Buparvaquone, a hydroxynaphtoquinone used for the treatment of bovine fever, have also demonstrated their action against important targets of the trypanosomatid energy metabolism. Initially, it was shown to be active against visceral leishmaniasis in BALB/c models [51,52]. This compound can inhibit cytochrome bc1 activity and decrease ATP levels of *L. (L.) donovani* and *L. (L.) mexicana* with reduced parasitic growth [53]. Moreover, Atavaquone (hydroxy-1,4-naphthoquinone) has been reported as an electron transport inhibitor in cytochrome bc1 in *Plasmodium* sp. [54]. We also emphasize that the action of naphthoquinone derivatives, possibly Lap and Law, on bioenergetic processes in mitochondria may involve other targets. [55,56].

**Figure 3:**
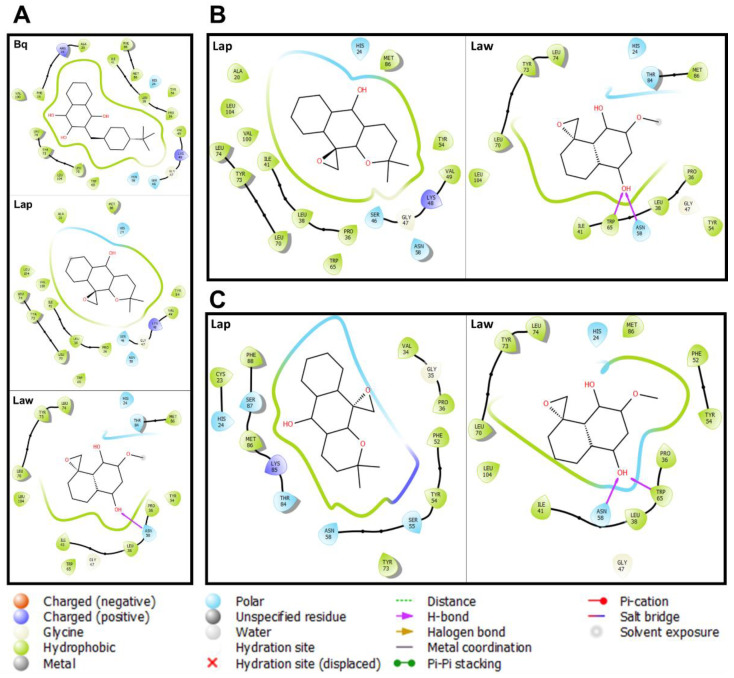
Molecular interaction of naphthoquinone derivatives with *Leishmania* spp. cytochrome c. Docking assays were performed with cytochrome c of *L.* (*L.*) *major* (**A**); *L.* (*L.*) *amazonensis* (**B**); and *L.* (*V.*) *braziliensis* (**C**). The 2D representation indicates the types of bonds that occur in cytochrome c with Buparvaquone (3-[(4-tert-butylcyclohexyl)methyl]-4-hydroxynaphthalene-1,2-dione-BH), Epoxy-α-lapachone (2,2-dimethyl-3,4-dihydrospiro[benzo[g]chromene-10,20-oxiran]-5(2H)-one-Lap), and Epoxymethyl-lawsone (2-Methyl-4H-spiro-[naphthalene-1,20-oxiran]-4-one-Law).

The molecular docking assays proposed here with cytochrome c are useful to assess the action potential of these compounds on the bioenergetics processes of *L. (V.) braziliensis* and *L. (L.) amazonensis*. Initially, the binding of cytochrome c was evaluated by comparing Burpavaquone with Lap and Law in *L. (L.) amazonensis* as a control approach. Burpavaquone binding profile was hydrophobic (*n* = 15), polar (*n* = 3), and positive charges (*n* = 2) amino acid residues mainly hydrophobic interactions. Comparatively, Lap presents a similar profile to Burpavaquone, only differentiated by the absence of two hydrophobic and one polar interaction. On the other hand, Law presented less bonds and the presence of a hydrogen bond with the amino acid residue ASN58 (Figure 3). The docking with the cytochrome c of *L. (V.) braziliensis* and *L. (L.) amazonensis* with Lap and Law reflects a different profile for each parasite species. In *L. (L.) amazonensis*, Lap presents a profile similar to the Buparvaquone, while in Law the same interactions were not identified, and it is possible to verify two hydrogen bonds: ASN 58 and TRP 65 (Figure 3). In *L. (V.) braziliensis*, the quantity of hydrophobic bonds decreases, with a greater contribution of polar bonds with Lap, while Law maintains the bond profile observed in *L. (L.) amazonensis*, differentiating only in the absence of interaction with the THR84 amino acid residue (Figure 3). Different from Burpavaquone, Lap and Law shows an interaction profile hydrophobic, positive charge, and polar for assessed *Leishmania* spp.

Other derivatives also seem to act as inhibitors of *P. falciparum* isoprenoid biosynthesis. This pathway is formed by menaquinone (vitamin K2) and α-tocopherol (vitamin E) employed in the electron carriage, in the mitochondrial respiratory chain, and protection the membranes against peroxidation, respectively [57]. These vitamins are derived from quinones, indicating a possible mechanism of action for hydroxynaphthoquinone based on their chemical core.

**Figure 4:**
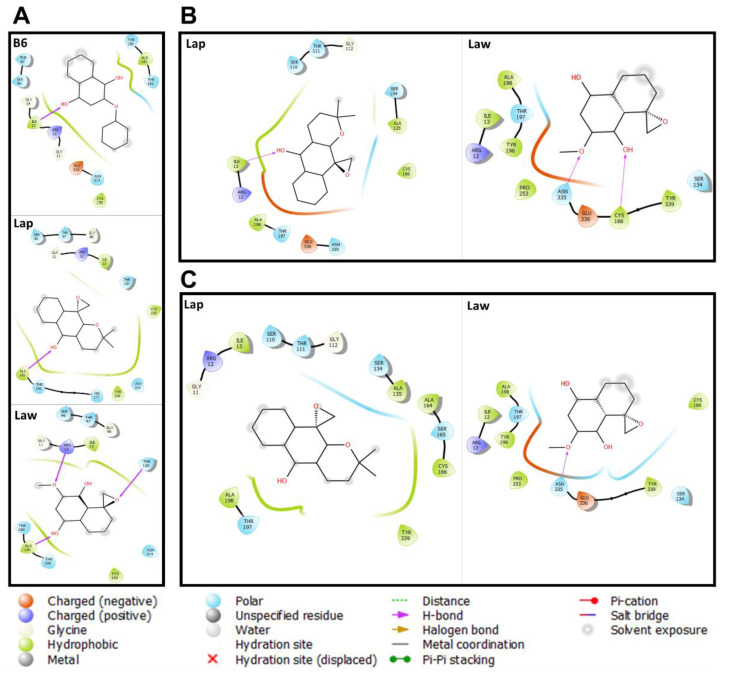
Molecular interaction of naphthoquinone derivatives with *Leishmania* spp. glyceraldehyde 3-phosphate dehydrogenase. Docking assays were performed with glyceraldehyde 3-phosphate dehydrogenase of *T. brucei* (**A**); *L.* (*L.*) *amazonensis* (**B**); and *L.* (*V.*) *braziliensis* (**C**). The 2D representation indicates the types of bonds that occur in glyceraldehyde 3-phosphate dehydrogenase with 2-phenoxy-1,4-naphthoquinone (B6), Epoxy-α-lapachone (2,2-dimethyl-3,4-dihydrospiro[benzo[g]chromene-10,20-oxiran]-5(2H)-one-Lap), and Epoxymethyl-lawsone (2-Methyl-4H-spiro-[naphthalene-1,20-oxiran]-4-one-Law).

Molecular details of the binding site of *T. brucei* glyceraldehyde-3-phosphate dehydrogenase with B6 showed polar (*n* = 5), hydrophobic (*n* = 3), and charged (*n* = 2) amino acid residues. In addition, a hydrogen bond was detected with the amino acid residue ILE13 and the hydroxyl of B6 that was a conserved bond in all structures crystallized with NAD ligand (Table S1). Comparatively, Lap and Law occupy the same B6 binding site, maintaining 83% and 75% of interactions with the amino acid residues of the binding site, respectively. However, there is a difference in the hydrogen bonds, where Law has three bonds with ARG12, THR120, and ALA181 residues and Lap maintains a hydroxyl bond with ALA181 (Figure 4). The way these compounds bind to the site of the *T. brucei* glyceraldehyde-3-phosphate dehydrogenase corroborated by the binding energy (≃−8.0 kcal/mol) of the interactions detected with the parasite enzyme.

The details of the molecular interactions of Lap and Law with the enzyme glyceraldehyde 3-phosphate dehydrogenase from *Leishmania* spp. were also assessed. In general, it was found that the interaction of Lap and Law showed differences in their binding mode according to the evaluated *Leishmania* spp. Law can bind to the enzyme site of both species with similar score value (−7.6 Kcal/mol), maintaining the binding characteristic: hydrophobic (*n* = 6), polar (*n* = 3), and charged (*n* = 2) amino acids, and hydrogen bonds (*n* = 2) (Figure 4). Lap binds differently in both species: *L. (L.) amazonensis* (hydrophobic (*n* = 4), polar (*n* = 5), and charged (*n* = 2) amino acids, and hydrogen bonds (*n* = 1)), and *L. (V.) braziliensis* (hydrophobic (*n* = 6), polar (*n* = 5), and charged (*n* = 1) amino acids). Though, Lap presents a closer binding score for both *Leishmania* spp., *L. (L.) amazonensis* (−8.5 Kcal/mol) and *L. (V.) braziliensis* (−8.3 Kcal/mol). Possibly, these findings may be related to the identity value (92%) between the enzymes of both species. Together, these findings are evidence that Lap and Law have the potential to inhibit the enzyme glyceraldehyde 3-phosphate dehydrogenase from *Leishmania* spp. Additionally, Lap and Law show a charge negative interaction profile when compared to B6 to the assessed *Leishmania* spp.

Currently, there are few effective pharmacological innovation actions for the treatment of leishmaniasis. A treatment based on drugs that act on more than one target of the parasite is a recommended option. This hypothesis was highlighted with the results presented in this work, as it reinforces the fact, from the point of view of the details of the molecular bond, that Lap and Law efficiently bind at acceptable energy levels in enzymes related to the parasite’s physiology such as cytochrome c, lanosterol C-14 demethylase, and glyceraldehyde-3-phosphate dehydrogenase.

Thus, the rational development of new chemotherapy based on functional interconnectivity between energy metabolism, electron transport chain, and lipid biosynthesis enzymes are an approach with high selectivity and effectiveness in the treatment of leishmaniases. In fact, Lap and Law compounds can act as a multi-target drugs, not only based on the results of this study, but also the set of mechanisms previously described in the literature [19,21,22]. These are potential and attractive compounds for new drugs, acting on more than one enzymatic target of *Leishmania* spp. Finally, their multi-target action might be a new promising strategy to treat these parasitic diseases using that approach, highlighting its advantages such as acting as network therapeutics blocking different and vital pathways, being less cytotoxic than the usual drugs, and might be able to act in complex diseases as leishmaniases and reducing drug resistance.

## 3. Materials and Methods

### 3.1. Chemical Structures

The chemical structures of the Epoxy-α-lapachone (CID: 12000280), Buparvaquone (CID: 71768), 2-phenoxy-1,4-naphthoquinone (CID: 15828698), and β-lapachone (CID: 3885) considered in this work were obtained in PubChem (https://pubchem.ncbi.nlm.nih.gov (accessed on 19 March 2021)). The Epoxymethyl-lawsone was obtained in the previous publication [21]. Cartesian structures coordinates were converted by the Avogadro software version 1.2 [58].

### 3.2. Protein Structures

In the first stage of this study, *Leishmania* spp. enzymes were selected from the TDRtarget v5 (http://tdrtargets.org/ (accessed on 1 October 2020)) database. Then, we evaluated the molecular interactions of naphthoquinone derivatives on glyceraldehyde-3-phosphate dehydrogenase (*T. brucei* (Tb927.6.4300), *L.* (*L.*) *amazonensis* (LAMA_000615100), and *L.* (*V.*) *braziliensis* (LbrM.30.2950)), cytochrome c from (*L.* (*L.*) *mexicana* (LmxM.16.1310), *L.* (*L.*) *amazonensis* (LAMA_000254700), and *L.* (*V.*) *braziliensis* (LbrM.16.1370)), and lanosterol C-14 demethylase from (*C. posadasii* (XP_003068142.1), *L.* (*L.*) *amazonensis* (LAMA_000181200.1), and *L.* (*V.*) *braziliensis* (LbrM.11.0880)). For this analysis, we used the crystal structures from the Protein Data Bank (https://www.rcsb.org/ (accessed on 19 November 2020)) and homologous structures of glyceraldehyde (code PDB: 1GYQ), cytochrome c (code PDB: 4DY9), and lanosterol C-14 demethylase (code PDB: 3L4D), were modeled by homology using Swiss-Model server (https://swissmodel.expasy.org/ (accessed on 3 December 2020)). Only the glyceraldehyde-3-phosphate dehydrogenase (code PDB: 4P8R) of *T. brucei* had its crystal structure deposited on Protein Databank. The sequences used were retrieved from TRITRYPDB and NCBI (https://tritrypdb.org/ (accessed on 10 December 2020) and https://www.ncbi.nlm.nih.gov/ (accessed on 10 December 2020)) and the selected templates have an identity of ≥50% with the target enzymes.

### 3.3. Prediction of the Interaction of Compounds on the Enzymes

The interaction of naphthoquinone derivatives with the receptors were determined by DockThor server [59]. These receptors and ligands were prepared with the MMFF94S force field to get the partial charge. The configuration of the grid box of each complex was determined by the redocking according to the reference ligand. This includes the definition of the center (average of the coordinates) and the size (20 Å in each dimension) of the grid box. The discretization was maintained at 0.25 Å. The redocking was validated with a root mean square of atomic positions deviation ≤2 Å.

## 4. Conclusions and Remarks

The results presented here contribute for enriching the elucidation of the possible mechanisms of action of two compounds derived from naphthoquinones, Lap and Law. Although, previous work has already demonstrated some possible mechanisms of action of Lap, here we bring more details about the action of this compound. Since Law is a derivative of the molecule α-lapachone and shares many characteristics with Lap, it was possible to predict common pathways between the two compounds. Thus, this work is pioneering in silico explanation of possible mechanisms of action of Law.

Data gathered here add molecular evidence of hit to the binding site of target enzymes of metabolic via: energy metabolism (glyceraldehyde-3-phosphate dehydrogenase), electron transport chain (cytochrome c), and lipid biosynthesis (lanosterol C-14 demethylase), reinforcing the mechanisms of action of both naphthoquinone derivatives in *Leishmania* spp. These in silico results corroborate with previous results described in the literature about the action of these compounds on enzymes of different biochemical pathways of the parasite pointing to the fact of the action of these compounds on multiple targets in *Leishmania* spp. This is certainly an advantage for a drug to effectively achieve leishmanicidal effects on the amastigote forms of the parasite during infection in the vertebrate host.

From the results, it is reinforcing that both compounds can bind in those *Leishmania* spp., as well as *T. brucei* enzymes. It is well known that these tropical neglected diseases are devastating, not only in their public health context but also for their psychological, cultural, and social stigma impacting patients’ lives. Other aspects are co-infection cases and parasitic burden after traditional treatment [60] as these parasites are successfully adapted to their host physiology. All of those treatment gaps remain in the current chemotherapy, but new approaches need to be developed focusing on disease control in strategic biochemical targets. As suggested here, Lap and Law have the potential to interact with key enzymes and potentially might be compounds used synergistically as new possible multi-target candidates for the treatment of different clinical forms of leishmaniases.

In addition, as leishmaniases are multifactorial diseases, using therapies directed for multiple targets can reduce drug-resistant and treatment-failure cases [61]. Drugs with multi-target characteristics are the latest pharmacological trend for the development of new therapies against leishmaniases [62]. Thus, compounds such as Lap and Law that showed a multi-target profile have the potential to interrupt the network of interactions between enzymes, which are vital to the parasite’s physiology.

## Figures and Tables

**Table 1:** Comparison of the docking energy (kcal/mol) of complexes from the assessed enzymes with ligands.

Parasites	Enzymes	Compounds				
		Lap	Law	B6	Buparvaquone	β-lapachone
*Coccidioides posadasii*	Lanosterol C-14 demethylase	−8.0	−8.0	ND	ND	−8.4
*Leishmania (L.) mexicana*	Cytochrome C	−10.2	−8.9	ND	−10.0	ND
*Trypanosoma brucei*	Glyceraldehyde-3-phosphate	−8.1	−7.5	−8.0	ND	ND
*Leishmania (L.) amazonensis*	Lanosterol C-14 demethylase	−8.4	−7.4	ND	ND	ND
	Cytochrome C	−10.0	−8.8	ND	ND	ND
	Glyceraldehyde-3-phosphate	−8.5	−7.6	ND	ND	ND
*Leishmania (V.) braziliensis*	Lanosterol C-14 demethylase	−8.2	−7.4	ND	ND	ND
	Cytochrome C	−9.0	−8.8	ND	ND	ND
	Glyceraldehyde-3-phosphate	−8.3	−7.7	ND	ND	ND

Lap: Epoxy-α-lapachone (2,2-dimethyl-3,4-dihydrospiro[benzo[g]chromene-10,20-oxiran]-5(2H)-one); Law: Epoxymethyl-lawsone (2-Methyl-4H-spiro-[naphthalene-1,20-oxiran]-4-one); B6: 2-phenoxy-1,4-naphthoquinone; ND: not determined.

## Data Availability

Data is contained within the communication.

## Supplementary Materials

The following is available online at. Figure S1: Inhibition of lipid biosynthesis in promastigotes, Table S1: Hydrogen bonds between trypanosomatid protein and their respective ligands.

## Appendix A

As molecular docking assays indicated the interaction of the enzyme lanosterol C-14 demethylase with epoxy-α-lapachone and epoxymethyl-lawsone, it was opportune to investigate the effect of compounds on the lipid biosynthesis. After incubation of the compounds with promastigotes, it was observed that both can interfere in the profile of lipids synthesized and acquired by the parasite. The chromatographic profile and arbitrary density values indicated that the untreated group presented intense lines corresponding to ergosterol and cholesterol, with less intense lines in not identified (NI1), probably accumulation of precursors in the pathway. The miconazole group (2 μΜ-reference control), already known to inhibit Lanosterol C-14 demethylase, showed a drastic inhibition of ergosterol and cholesterol with considerable accumulation of precursors in the NI1 and NI2 lines (Figure S1). In the group treated with epoxymethyl-lawsone (16.3 μΜ) there was a marked loss of acquired cholesterol, and a decrease in ergosterol synthesis, with a considerable accumulation of pathway precursors in NI1. In epoxy-α-lapachone group (6.3 μΜ), the acquired cholesterol remained close to the control, in contrast to the ergosterol synthesis, which was reduced by almost half in comparison to the untreated group, and with values approximately to the miconazole group. Since the final product of the pathway is not synthesized, precursor products must be observed more intensely, as occurred in epoxymethyl-lawsone (NI1). The NI3 line was observed by densitometry only in epoxymethyl-lawsone, however, possibly an accumulation of cholesterol.

This simple assay demonstrates the real interference of oxirans derived from natural naphthoquinones in the lipid synthesis of the *Leishmania* promastigote membrane by inhibiting the enzyme lanosterol C-14 demethylase.
