# Supplementary material for: In Silico Insights into the Mechanism of Action of Epoxy-α-Lapachone and Epoxymethyl-Lawsone in Leishmania spp
Source: Molecules. 2021 Jun 10;26(12):3537. doi: 10.3390/molecules26123537 (PMC8229338; doi:10.3390/molecules26123537)
Supplement: Supplementary file 1 [file molecules-26-03537-s001.zip › molecules-1178413-supplementary.pdf]

Supplementary files

Table 1. Hydrogen bonds between trypanosomatid protein and their respective ligands.

Glyceraldehyde-3-Phosphate Dehydrogenase						Cytochrome c				Lanosterol-14-alpha demetilase					
	Protein ligand complex (NAD*)					Protein ligand complex (HEME**)				Protein ligand complex (HEME**)					
	1GYP	1GYQ***	3DMT	1QXS	4P8R		4DY9	4GED	3RIV		2X2N	2WV2	2WVZ	3L4D	3P99
	Number of binding						Number of binding				Number of binding				
Phe10	-	-	1	-	-	His29	1	1	-	Tyr103	1	1	1	-	1
Arg12	1	1	1	-	1	Gly52	1	-	-	Tyr102	-	-	-	1	-
Ile13	1	1	1	1	1	Tyr59	1	-	-	Tyr115	-	-	-	1	-
Asp38	1	1	1	1	-	Ser60	1	-	-	Tyr116	1	1	1	-	-
Arg78	-	-	-	-	1	Asn63	1	-	-	Arg123	-	-	-	1	-
Gln91	-	1	1	1	-	Trp70	1	-	-	Arg124	1	1	1	-	1
Trh120	-	-	-	-	1	Lys90	1	-	-	Ala221	1	-	-	-	-
Ser134	1	1	1	-	-	Met91	1	1	-	Arg360	-	-	-	1	-
Asn335	-	1	1	-	-	His192	-	-	1	Arg361	1	1	1	-	1
						Glu196	-	-	1	His420	1	1	1	1	-
						His198	-	-	1	Cys422	1	1	1	-	1
						Ser202	-	-	1						

* Nicotinamide-adenine-dinucleotide; ** Protoporphyrin ix containing Fe and *** N6-benzyl-nicotinamide-adenine-dinucleotide

Supplementary files

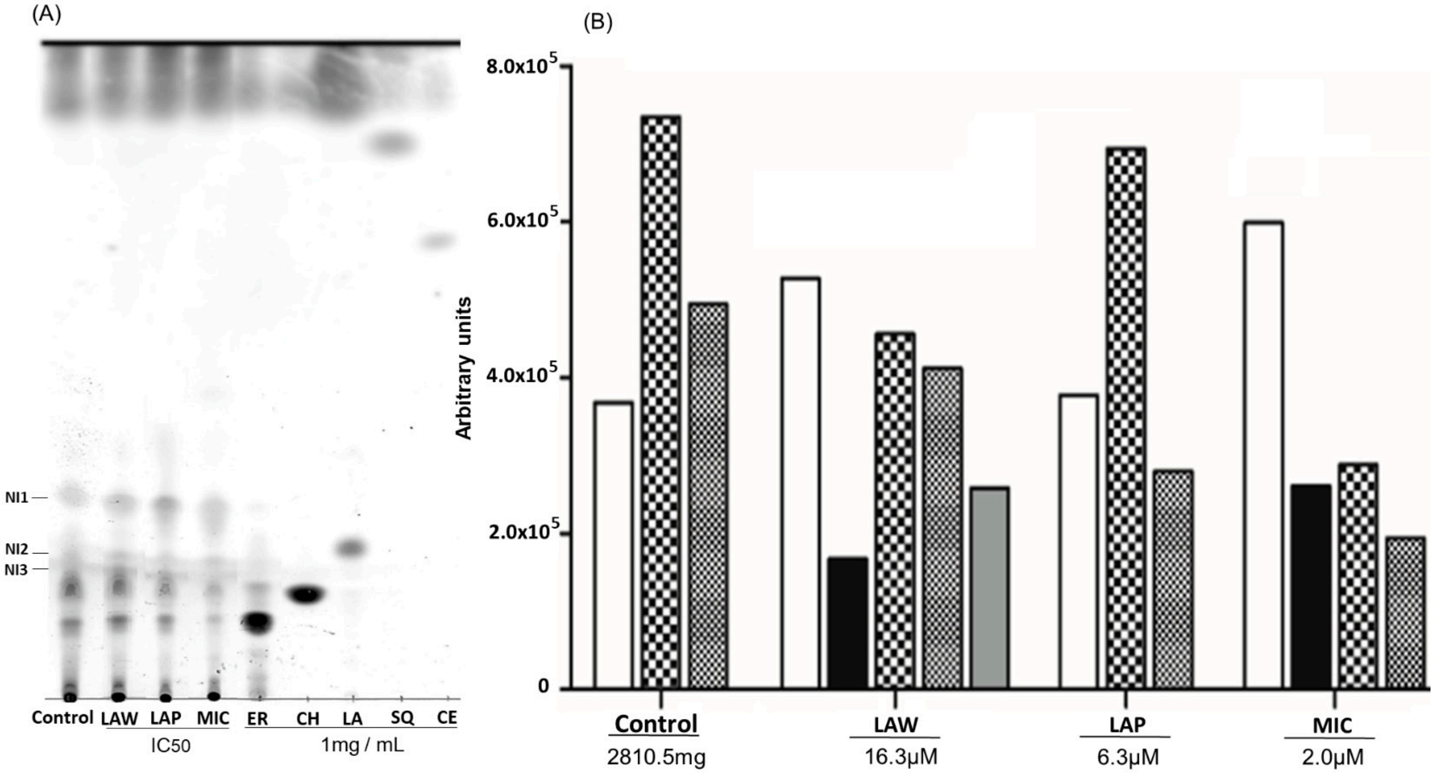

Figure S1. Inhibition of lipid biosynthesis in promastigotes. (**A**) Effect of oxyranes on the biosynthesis of *L.* (*V.*) *braziliensis* promastigote ergosterol: Lipid extracts of 2810.5 ± 208 mg (fresh weight) of the promastigotes not incubated to the compounds (Control); incubated with IC50 values of epoxymethyl-lawsone (LAW = 16.3μM), epoxy-α-lapachone (LAP = 6.3 μM), miconazole (MIC = 2.0 μM), and commercial ergosterol standards (ER = 1 mg/mL); cholesterol (CH = 1 mg/mL), lanosterol (LA = 1 mg/mL), squalene (SQ = 1 mg/mL), and cholesterol ester (CE = 1 mg/mL) were evaluated by Thin layer chromatography (TLC) for 24 h. After the chromatography tests, the lipid profiles were developed using chromatography sheet with "charring" solution. Not identified (NI). (**B**) The quantification of *L.* (*V.*) *braziliensis* promastigote sterols was obtained according to arbitrary values of the density of the lanes, using the imageMaster program (version 1.11). Not identified 1 (NI1 ▭); Not identified 2; (NI2 ■); Not identified 3 (NI3 ▭); ergosterol (▦); cholesterol (▦); epoxymethyl-lawsone (LAW), epoxy-α-lapachone (LAP), miconazole (MIC).
